# Impact of Hashimoto’s thyroiditis on ultrasound diagnosis of papillary thyroid carcinoma: a retrospective study

**DOI:** 10.3389/fonc.2025.1551114

**Published:** 2025-05-15

**Authors:** Chao Teng, Kunkun Pang, Lulu Zhang, Yuan Li, Xiuliang Wei, Feixue Zhang

**Affiliations:** ^1^ Department of Traditional Chinese Medicine, The Second Hospital of Shandong University, Jinan, Shandong, China; ^2^ Department of Ultrasound, The Second Hospital of Shandong University, Jinan, Shandong, China; ^3^ Department of Pathology, The Second Hospital of Shandong University, Jinan, Shandong, China

**Keywords:** thyroid, Hashimoto’s thyroiditis, papillary thyroid carcinoma, ultrasound diagnosis, ultrasound

## Abstract

**Objective:**

To investigate the clinical features and ultrasonic manifestations of papillary thyroid carcinoma (PTC) with Hashimoto’s thyroiditis (HT) and without HT. The characteristics were analyzed to improve the accuracy of diagnosing PTC with HT via ultrasound, potentially reducing overtreatment in certain cases.

**Methods:**

The patients were retrospectively analyzed in the Second Hospital of Shandong University from December 2015 to January 2020. A total of 5732 patients had thyroid nodules on ultrasound. Among them, 553 patients (702 nodules) received surgical treatment and the histopathological results showed PTC with or without HT were enrolled. Univariate and multivariate analyses were conducted to evaluate the risk factors associated with HT influencing the ultrasound diagnosis of PTC. The nodules were categorized into two groups based on pathological results: PTC with HT and PTC without HT.

**Results:**

Gender, nodule echo, posterior echo change, border, aspect ratio, and nodule invasion rate significantly differed between the two groups (*P*<0.05). Independent variables for the multivariate logistic regression model were selected from those that showed statistical significance (*P*<0.05) in the univariate analysis. The results showed that the model was statistically significant (*χ^2^ =* 4.717, *P*<0.001, *R^2^ =* 0.185). Being female and aspect ratio ≥1 were identified as the risk factors for the diagnosis of PTC with HT, and the values were higher in group A than in group B by 3.15 and 1.73, respectively (OR=3.15, 1.73, *P*<0.05). Moreover, HT was identified as the protective factor because PTC was less likely to invade the thyroid capsule than the control group (OR=0.47, *P*<0.05).

**Conclusions:**

HT can affect the clinical and ultrasonographic features of PTC in distinct ways. It provides a protective effect on the capsule, significantly reducing capsular invasion, while female gender and an aspect ratio ≥1 are associated with an increased risk of PTC with HT diagnosis.

## 1 Introduction

Hashimoto’s thyroiditis (HT), is a chronic thyroid gland of inflammation and the most common cause of inflammatory diseases leading to hypothyroidism (1, 2). Papillary thyroid carcinoma (PTC) is the most common subtype of thyroid cancer. The incidence of PTC is rapidly increasing worldwide, possibly because of the improvement of auxiliary screening methods (3). However, the relationship between HT and PTC is controversial (3–5). Researchers have postulated that HT is a risk factor promoting PTC development (4, 6), possibly because of the increased risk of some tumors in patients with autoimmune diseases, leading to frequent tumor transformation (7, 8). Other scholars believe that HT is a protective factor that reduces the incidence of PTC and prevents metastasis and recurrence (9, 10). Based on a literature review (4, 6–10), we found that few studies have explored the ultrasound imaging characteristics of PTC with Hashimoto’s thyroiditis. Therefore, our study can supplement this research and explore the relationship between PTC and HT from the perspective of ultrasound imaging.

Although ultrasound has revolutionized the screening for PTC, the role of ultrasound in diagnosing PTC with HT background remain to be a subject of debate in recent years (11). Baser H et al. showed that HT cannot affect the characteristics of PTC ultrasound images (4). However, Singh B et al. indicated that HT reduces the preoperative diagnosis rate of ultrasound for PTC (12). Several sonographic features of thyroid nodules (ill-defined, irregular morphology, hypoechoic, microcalcifications, and aspect ratio) are correlated with PTC. HT has complex and variable ultrasonographic manifestations, including numerous micronodules (size: 1.5 mm-3 mm) in the fibrous, enlarged, and lobulated glandular parenchyma, and diffused localized regions of low echo separated by the high echo of the strip (4, 13, 14). The heterogeneous glandular architecture associated with HT can obscure the distinction between benign and malignant nodules, potentially leading to diagnostic bias during ultrasound evaluation (4). In this study, the clinical and ultrasonographic features of pathologically-confirmed PTC with HT and without HT were retrospectively investigated to further explore the effect of HT background on the ultrasonic diagnosis of PTC.

## 2 Materials and methods

### 2.1 General material

**Figure 1:**
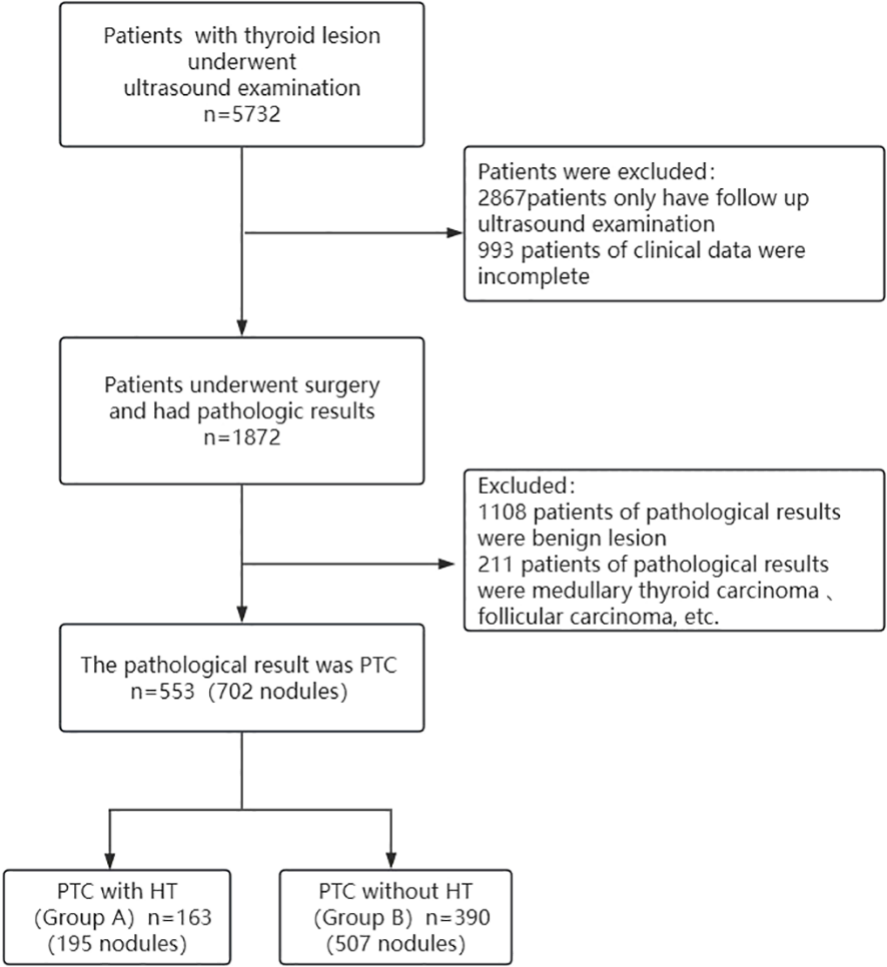
The flowchart showing patient selection protocol.

**Figure 2:**
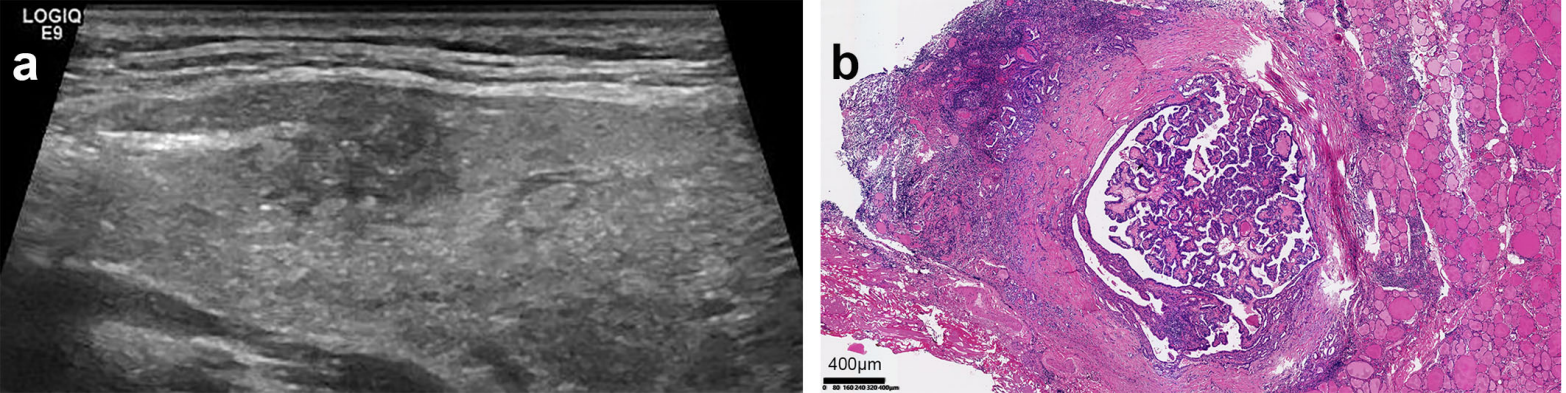
Ultrasound and pathological images of PTC with HT. **(a)** The parenchymal echo of the thyroid gland was thickened and less homogeneous. The node exhibited the following sonographic features: a discontinuous ventral dorsal membrane anteriorly, scattered punctate hyperechoic foci, heterogeneous internal echogenicity, irregular margins, and poorly defined borders. **(b)** Pathology result: (HE, x40)papillary thyroid carcinoma infiltrating the capsule, accompanied by Hashimoto’s disease changes in the surrounding thyroid.

**Figure 3:**
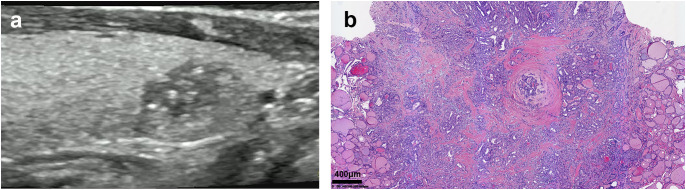
Ultrasound and pathological images of PTC without HT. **(a)** The parenchymal echo of the thyroid gland was homogeneous, and a hypoechoic nodule was non-homogeneous internal echo, and a dotted strong echo. **(b)** Pathology result: (HE, x40)Papillary thyroid carcinoma.

A total of 553 patients with thyroid lesions were retrospectively analyzed in Second Hospital of Shandong University from December 2015 to January 2020. Inclusion criteria: ① The patients were diagnosed with thyroid nodules via Ultrasound; ② They underwent surgery; ③ The histopathological findings were PTC. Exclusion criteria: ① The clinical data were incomplete; ② The histopathological findings were results were benign lesion, medullary thyroid carcinoma, follicular carcinoma, etc. Histopathology is the gold standard for diagnosis, HT and PTC are determined by histopathology. A flowchart showing patient selection protocol is presented in **Figure 1**. Hematoxylin and eosin (H&E) staining was performed according to routine procedure. The sections were mounted and observed under light microscopy. The magnification was 40x. The lesions were divided into two groups based on the pathological results PTC with HT (group A)(**Figures 2a, b**)and PTC without HT (group B) (**Figures 3a, b**). The pathological results of HT showed diffuse thyroid lymphocytic infiltration, lymphoid follicle formation, and atrophic fibrosis of the thyroid parenchyma (15, 16). The clinical characteristics of patients were obtained from outpatient/inpatient electronic medical record systems. The ultrasound image data were obtained from an ultrasound imaging workstation.

### 2.2 Inspection method

Following scanning of the thyroid and cervical lymph nodes were scanned, and the lesions were carefully identified using GE Logic E9 color Doppler ultrasound diagnostic apparatus, linear array probe, and probe frequency of 9~15MHz. The lesions were observed through multi-section and multi-angle. The relevant data and images were measured and recorded. The images were analyzed by two sonographers blinded to the pathological results (more than three years of experience). The location, number, size, boundary, internal echo and echo homogeneity of the lesion, posterior echo change, calcification characteristics, relationship with the membrane, and blood flow signals were assessed. The sonographers negotiated with each other and reached a consensus whenever a disagreement occurred. The blood flow signal was displayed in the lesion using color Doppler. The blood flow signal was classified into four grades using the Adler semi-quantitative method (17): grade 0: no blood flow in the lesion; grade I: low blood supply, only 1–2 punctate or short rod-shaped blood flow signals; grade II: moderate blood flow with one long or 3–4 punctate blood vessels; grade III: abundant blood flow with two long and five or more punctate blood vessels.

### 2.3 Statistical methods

All data were analyzed using the SPSS 20.0 software. Normally distributed measurement data were expressed as mean ± standard deviation ( $\bar{x}$ ± *s*). Otherwise, the data were expressed as median (quartile). An appropriate statistical analysis method was selected using univariate analysis based on the type of independent variables: independent samples t-test for normally distributed numerical variables, nonparametric test (Mann Whitney U test) for non-normally distributed data, Pearson chi-square test or Fisher’s exact test for categorical variables. The statistically significant variables (*P*<0.05) in the univariate analysis were included in the multivariate analysis model.

## 3 Results

### 3.1 Clinical data

The overall study population consisted of 553 individuals (445 female, 108 male), with ages ranging from 15 to 82 years (mean ± SD: 46.1 ± 11.9 years). Group A comprised 163 PTC patients (mean age: 45.2 ± 0.9 years), while Group B included 390 patients (mean age: 46.5 ± 0.6 years). The average ages were not significantly different between the two groups (*U*=1.149, *P*=0.251). Groups A and B had 150 (92.0%) and 295 (75.6%) female patients, respectively, (*X^2^ =* 19.634, *P*<0.001). Pathology results showed that 52 cases (31.3%) in group A and 117 cases (45.7%) in group B had lymph node metastasis (*X^2^ =* 9.427, *P*=0.002).

### 3.2 Ultrasound features

A total of 195 nodules were detected in group A (length: 0.98 ± 0.06 cm; width: 0.75 ± 0.05 cm; and thickness: 0.69 ± 0.04 cm), while 507 nodules (length: 0.97 ± 0.04 cm; width: 0.77 ± 0.03cm; and thickness: 0.72 ± 0.03 cm) were detected in group B. The length, width, thickness, volume, position, edge, shape, calcification characteristics, and blood flow characteristics of the nodules were not significantly different between the two groups (*P*>0.05). However, nodule echo, posterior echo change, boundary, aspect ratio and nodule invasion rate were significantly different between the two groups (*P*<0.05). A total of 27 cases (16.6%) in group A and 89 cases (22.8%) in group B had multiple lesions (*X^2^ =* 2.714, *P*=0.099). The above results are shown in **Table 1**.

**Table 1:** Ultrasound features of PTC with HT or without HT.

Ultrasound feature		Group A (n=195)	Group B (n=507)	T/U/χ^2^	*P*
Length(cm)		0.98±0.06	0.97±0.04	0.026	0.979
Width(cm)		0.75±0.05	0.77±0.03	0.325	0.745
Thickness(cm)		0.69±0.04	0.72±0.03	0.611	0.541
Volume(cm^3^)		0.96±0.03	1.21±0.03	0.501	0.617
Location	Left	103 (52.8%)	240 (47.3%)	2.434	0.296
	Right	87 (44.6%)	258 (50.9%)		
	Isthmus	5 (2.6%)	9 (1.8%)		
Echo	Hypoecho	182 (93.3%)	498 (98.2%)	14.021	**<0.001**
	Isoecho/hyperecho	10 (5.2%)	4 (0.8%)		
	Cystic and solid	3 (1.5%)	5 (1.0%)		
Posterior echo	Uniformity	160 (82.1%)	477 (94.1%)	24.924	**<0.001**
	Attenuation	34 (17.4%)	28 (5.5%)		
	Enhancement	1 (0.5%)	2 (0.4%)		
Boundary	Distinct Indistinct	71 (36.4%) 124 (63.6%)	114 (22.5%) 393 (77.5%)	14.071	**<0.001**
Edge	Smooth	134 (68.7%)	366 (72.2%)	0.828	0.363
	Unsmooth	61 (31.3%)	141 (27.8%)		
Shape	Regular Irregular	53 (27.2%) 142 (72.8%)	145 (28.6%) 362 (71.4%)	0.140	0.708
Aspect ratio	<1	63 (32.3%)	213 (42.0%)	5.559	**0.018**
	≥1	132 (67.7%)	294 (58.0%)		
Calcification	No	60 (30.8%)	174 (34.3%)	1.157	0.561
	Macrocalcification	11 (5.6%)	33 (6.5%)		
	Microcalcification	12 (63.6%)	300 (59.2%)		
Capsular invasion	No	174 (89.2%)	402 (79.3%)	7.044	**0.002**
	Have	21 (10.8%)	105 (20.7%)		
CDFI	0	153 (78.5%)	397 (78.3%)	7.191	0.066
	I	14 (7.1%)	18 (3.5%)		
	II	12 (6.2%)	54 (10.7%)		
	III	16 (8.2%)	38 (7.5%)		

Bold:P<0.05.

### 3.3 Ultrasound diagnosis results

Ultrasound misdiagnosed 54 (27.7%) of nodules with HT and 100 (19.7%) of nodules without HT as benign. Further analysis found that the misdiagnosis rate was significantly higher in the HT group than in the non-HT group (*χ^2^ =* 5.222, *P*=0.022).

### 3.4 Multivariate logistic regression analysis

Variables that were significant in univariate analysis (*P*<0.05) were included in multivariate logistic regression analysis as independent variables. The results showed that the model was statistically significant (*χ^2^ =* 4.717, *P*<0.001, *R^2^ =* 0.185). Female patients with HT and nodule aspect ratio ≥1 were risk factors, which were 3.15 and 1.73 times more likely to be diagnosed with PTC than those without HT.

HT was identified as a protective factor in patients with PTC, significantly reducing the likelihood of thyroid capsule invasion. Patients with coexisting HT had a 47% lower risk of capsular invasion compared to those without HT. The above results are shown in **Table 2**.

**Table 2:** Multivariate logistic regression analysis.

Variables	*β*	OR(95%CI)	*P*
Sex	1.149	3.15(1.66-5.98)	**<0.001**
Aspect ratio	0.548	1.73(1.12-2.68)	**0.014**
Capsular invasion	-0.755	0.47(0.26-0.86)	**0.014**

Model: χ^2^=4.717, P<0.001, R^2^ =0.185.

Bold:P<0.05.

## 4 Discussion

The association of HT with PTC from different aspects (clinical, pathological, gene expression, biomarkers, and ultrasonographic features) has been investigated in numerous studies since it was first proposed by Dailey et al. in 1955 (18). Some scholars believe that HT is a risk factor that promotes PTC progression. Although some studies suggest that HT may lower the risk of lymph node metastasis and recurrence in PTC, contributing to a more favorable prognosis, other research reports no significant association between tumor characteristics, such as nodule size and multifocality, and PTC outcomes. In this study, the misdiagnosis rate of ultrasonography was significantly higher in group A than in group B (27.7% *vs*. 19.7%) (*P*=0.022). This result suggests that HT can affect the clinical and ultrasonic characteristics of PTC nodules, thus influencing the accuracy of ultrasonic diagnosis.

Moreover, 195 of 702 PTCs were associated with HT (incidence rate: 27.8%), similar to the findings of Liang et al. (19). Liang et al. suggested that female gender, age, tumor size, and multifocal lesions may influence PTC development and lymph node metastasis, thus can predict a good prognosis (19). Other studies have also shown that these factors affect lymph node metastasis and capsular invasion (4). Our findings align with previous studies regarding female predominance, capsular invasion, and lymph node metastasis. However, there are variations in age, tumor size, and multifocality.

Although HT and PTC can occur at any age, thyroid cancer is common among patients aged 30–40 years, especially in female patients (20). In this study, the mean age at onset of PTC was not significantly different between the two groups (45.2 ± 0.9years in group A and 46.5 ± 0.6 years in group B) (*P*=0.251), inconsistent with previous studies (21, 22). Furthermore, the incidence rate of PTC was higher in women with HT than in women without HT (92.0% *vs*. 75.6%). The number of female patients with PTC was significantly different between the two groups (OR=1.149, *P*<0.001), which is consistent with findings from previous studies (22). This finding indicates that female patients have a higher likelihood of developing PTC, suggesting that female gender may be a potential risk factor for PTC patients with HT.

PTC nodules in group A exhibited a lower likelihood of capsular invasion, with a significantly different invasion rate between the groups (OR=0.47, *P*=0.014), aligning with findings from previous studies (23, 24). This difference could be because of: First, the invasiveness of PTC nodules with HT is lower than that of PTC patients without HT (protective mechanism), resulting in less invasion; Second, the specific location of the pure PTC nodule. A nodule is more likely to invade the capsule if it is closer to the capsule. However, the distance between each nodule and capsule was not analyzed here; thus, further research is warranted.

In this study, the cervical lymph node metastasis rate was significantly lower in group A than in group B (31.3% *vs*. 45.7%) (*χ^2^ =* 9.427, *P*= 0.002). Liang et al. showed that lymph node metastasis rate is significantly lower in PTC patients with HT than that in PTC patients without HT, consistent with this study (19). This may be due to the presence of multiple thyroid antibodies in the thyroid tissue of PTC patients with Hashimoto’s thyroiditis (HT). The immune response triggered by these antibodies may contribute to tumor cell destruction or confinement, thereby exerting a protective effect in these patients (1, 19).

Moreover, boundary, echo and posterior echo changes were significantly different between the two groups (*P*<0.05). In contrast, border and shape were not significantly different between the two groups (*P*>0.05). These findings differ from previous studies (4, 13, 25), suggesting that HT influences the ultrasound characteristics of PTC in a distinct manner. One possible explanation is the prolonged disease course of HT, which leads to diverse pathological changes. Moreover, concurrent malignant nodules are usually in the subclinical stage with few characteristics of malignant lesions; Second, demographic characteristics, environmental factors, sample size, and multi-center or single-center studies may affect different research results, resulting in inconsistencies.

Aspect ratio is one of the most specific and sensitive indicators of PTC, providing assessment of tumor growth (26, 27). Aspect ratio refers to the vertical diameter/horizontal diameter of the nodule. Vertical diameter refers to the maximum anterior-to-posterior diameter of the nodule perpendicular to the skin. Horizontal diameter is defined as the maximum diameter of the nodule parallel to the skin. In the early growth pattern of PTC, the diameter lines are more in the vertical diameters of the tumor than in the horizontal diameters (aspect ratio≥1). This may be attributed to the preferential distribution of cancer cells along the anterior-posterior axis during the tumor’s proliferative phase, whereas in other stages, cancer cells tend to disperse more broadly in different directions (28). In this study, 132 (67.7%) nodules in the HT group and 294 (58.0%) nodules in the non-HT group had an aspect ratio of ≥1 (OR=1.73, *P*=0.014). This difference may be attributed to the more frequent use of ultrasonography in patients with HT, which facilitates earlier detection and diagnosis of abnormal nodules associated with the condition (1, 29).

Sand-like micro-calcification is widely used as ultrasound malignancy markers of PTC and has high specificity and positive predictive value (30). However, Ohmori et al. (13) found that PTC patients with HT present with macrocalcifications and fewer sand-like microcalcifications. In this study, the calcification characteristics of nodules were not significantly different between the two groups (*P*=0.561). The proportions of macrocalcifications and microcalcifications were also comparable between the results(5.6% *vs*. 6.5%; 63.6% *vs*. 59.2%), inconsistent with previous studies (13). Therefore, further studies should investigate the influence of HT on calcification formation.

However, this study has some limitations. First, this was a single-center retrospective study with a small sample. Therefore, there may be some data collection biases, which could potentially affect the results. Second, some conclusions are inconsistent with previous studies, and thus a multi-center study with large sample is needed to verify the results.

In conclusion, HT can affect the clinical and sonographic features of PTC. Female gender and aspect ratio≥1 have a higher chance of developing PTC. However, HT can confer protection in PTC patients by preventing capsule invasion. To validate these results, we will conduct a prospective study for patients diagnosed with PTC through ultrasound with or without HT.

## Data Availability

The original contributions presented in the study are included in the article/Supplementary Material. Further inquiries can be directed to the corresponding authors.
